# Unlocking Self-Esteem in Older Adults: A Conceptual Exploration of Technological Proficiency and Its Effects

**DOI:** 10.3390/bs15030306

**Published:** 2025-03-04

**Authors:** Wenjie Zhu, Nurul Hidayu Mat Jusoh, Ribka Alan, Malisah Latip, Juniza Md Saad

**Affiliations:** Faculty of Humanities Management and Science, Universiti Putra Malaysia Bintulu Sarawak Campus, Bintulu 97008, Malaysia; nurulhidayu@upm.edu.my (N.H.M.J.); ribka@upm.edu.my (R.A.); malisah@upm.edu.my (M.L.); juniza@upm.edu.my (J.M.S.)

**Keywords:** technological proficiency, self-esteem, older adults

## Abstract

With the current rapid increase in digital technology adoption, understanding the relationship between technological proficiency and self-esteem is crucial to older adults. This study explores the mechanisms through which technological proficiency influences self-esteem, particularly considering its associations with technological engagement, self-efficacy, social participation, and autonomy. By using a narrative review approach, this study synthesizes recent findings from gerontology and psychology to examine these relationships. The analysis shows that higher technological proficiency enhances self-esteem by boosting self-efficacy, fostering social participation, and promoting greater autonomy. Older adults who are skilled in using digital tools exhibit greater confidence in managing daily activities and maintaining their social networks, contributing to their psychological resilience. Conversely, lower technological proficiency is linked to frustration, social exclusion, and diminished self-esteem, exacerbated by the digital divide. However, despite these insights, much remains unexplored; for example, no longitudinal study has been conducted to capture the dynamic relations between technological proficiency and self-esteem. These findings suggest that further research is needed to learn more about how technological proficiency influences self-esteem and recommend clinical ways of supporting older adults in their current stage.

## 1. Introduction

Aging populations are expanding rapidly, with the global population aged 60 years and over expected to double to 2.1 billion by 2050. As societies become increasingly digital, older adults must adapt to new technologies that are now integral to communication, healthcare, and daily activities. While these innovations offer many advantages, they also require continuous learning, which can be challenging for those with limited digital experience. Older adults who struggle to keep pace with technological advancements may face frustration, social exclusion, and a diminished sense of autonomy, all of which can negatively impact their self-esteem and psychological well-being. The difficulties with adapting to digital technological advancements may affect older adults’ quality of life, especially when they must incorporate technologies into everyday life. Such difficulties can take a toll on their self-esteem and affect their psychological well-being. Despite the numerous benefits provided by digital technology, such as greater access to information, multiple channels of communication, and the simplification of everyday chores, a high percentage of older adults still face significant difficulties in operating digital tools and media such as smart phones, e-commerce banking, and social media. According to Iancu and Iancu (2020), the difference between the technological abilities of the young and the older generation might lead to older adults’ isolation and marginalization. For instance, the older age may face challenges in accessing services, maintaining social networks, or feelings of alienation from younger members of their family who are more conversant with modern technology. Such a digital divide can obviously promote a decline in self-esteem and increase feelings of inadequacy or dependency. Guner and Acarturk (2020) state that while older adults are aware of the benefits of technology, they need support, encouragement, and friendlier interfaces to access them. The lack of attention older adults pay to new technology deepens the digital divide, increases perceptions of loneliness, limits their independence, and ultimately lowers their self-esteem. Guner and Acarturk’ research highlights the need for developing accessible and usable technology to promote health and happiness among elderly groups.

As the recognition of the digital divide among older adults grows, there is still a high demand for research on the connection between technological proficiency and self-esteem. While existing research has explored the psychological implications tied with technology usage and competency (Haidt & Allen, 2020), research on the connection between technological proficiency and self-esteem among older-aged population is still in its infancy (Herlina et al., 2024). The current research study aims to offer a systematic literature review on the topic and recommend potential future research pathways on the connection between technological proficiency and the older-aged population. In achieving this purpose, the literature review aims to better inform our understanding of aging-related issues, technology usage, and psychological health and offer insights into how technological proficiency could be enhanced and how the negative implications resulting from a lack of technological proficiency among older adults could be reduced.

The primary research aim is to examine the connection between technological proficiency and self-esteem in older adults. More specifically, the study aims to gain a better understanding of how varying levels of technological proficiency affect psychological health in reference to constructs such as self-efficacy, societal integration, and generalized trust in technology situations. It is expected that this research study will reveal substantial pathways underlying how technological proficiency contributes to increasing self-esteem, especially in reducing social isolation, increasing digital independence, and promoting self-efficacy in older adults. In addition, this research study aims to identify potential barriers to technology access and usage among older adults.

## 2. Methods

This study provides a narrative overview of the available literature on the relationship between technological proficiency and older adults’ self-esteem. In contrast to systematic reviews, which are required to adhere strictly to certain standards such as PRISMA (Preferred Reporting Items for Systematic Reviews and Meta-Analyses) (Green et al., 2006), the use of a narrative review approach allows for the iterative refinement of the search terms and inclusion criteria while maintaining coherence in the selection of relevant studies (Byrne, 2016). The methodological structure of a narrative overview synthesizes diverse views based on psychological, sociological, and technological research with a view toward developing a comprehensive understanding of the topic. The high availability of peer-reviewed literature works had a major impact on database choice and accessibility. The “cited by” feature on Google Scholar was also useful in tracking down other relevant scholarly studies, while Scopus provided a curated database with a systematic and comprehensive literature review process enhancement (Ferrari, 2015). Studies from the past six years (2018–2023) were prioritized in an attempt to focus the review on contemporary findings.

The initial search utilized broad keywords, including “Self-Esteem”, “Technological Proficiency”, “Older Adults”, and “Technology Acceptance”, combined by using Boolean operators such as “AND” and “OR” to refine results. Preliminary searches guided the addition of terms such as “Digital Divide”, “Social Inclusion”, and “Autonomy in Aging” to expand the scope of this review. Articles were included if they addressed older adults’ engagement with technology and its psychological impact, particularly on self-esteem. Empirical research provided evidence-supported results, narrative overviews synthesized the relevant literature, and metanalyses provided the numerical validation of broad trends. To maintain consistency and credibility, only studies written in the English language and meeting the inclusion criteria were included. Exclusions were applied to studies focusing exclusively on younger populations or workplace technology use and studies lacking sufficient methodological rigor. Studies that examined broader psychological outcomes of technology use but did not specifically consider self-esteem were excluded unless they provided valuable theoretical frameworks or insights.

The search process emphasized breadth and global representation, incorporating studies from regions such as North America, Europe, and Asia, with the majority of studies originating from the United States and Europe. Although the focus was on recent studies, no strict time restrictions were applied, allowing for the inclusion of highly cited foundational works. Data extraction involved categorizing articles under key themes, including “Technological Proficiency and Self-Esteem”, “Barriers to Digital Engagement”, and “Psychological Outcomes of Technology Use”. Each selected article was systematically analyzed based on its research objectives, methodology, key findings, and implications. Particular attention was given to the role of technological proficiency in fostering autonomy, social inclusion, and psychological resilience among older adults. Supplementary searches were conducted by hand-searching references from key articles, ensuring comprehensive coverage of the topic. The final selection consisted of 50 peer-reviewed articles, reflecting diverse methodologies and perspectives.

While this narrative review offers the advantage of flexibility and breadth, it is inherently less structured than systematic reviews, making it susceptible to selection bias. Additionally, the reliance on Google Scholar may have limited access to domain-specific databases. However, these limitations are counterbalanced by the comprehensive scope and iterative refinement of search strategies, which allowed for the integration of diverse insights into the relationship between technological proficiency and self-esteem among older adults.

## 3. Results

### 3.1. Article Selection

The literature search was conducted across Google Scholar and Scopus, yielding a total of 913 articles (632 from Google Scholar and 281 from Scopus). To ensure relevance and methodological rigor, a multi-stage selection process was implemented, involving duplicate removal, title and abstract screening, and full-text eligibility assessment.

In the first stage, duplicate records were identified and removed, eliminating 562 articles and leaving 351 articles for further evaluation. The remaining articles were then screened based on their titles and abstracts to determine their relevance to the research topic. In this stage, 301 articles were excluded. The primary reasons for exclusion included studies focusing on younger individuals rather than older adults (*n* = 221), articles that discussed general digital skills without directly addressing technological proficiency in relation to self-esteem (*n* = 62), and studies that were published in languages other than English, making them inaccessible for analysis (*n* = 18).

**Figure 1:**
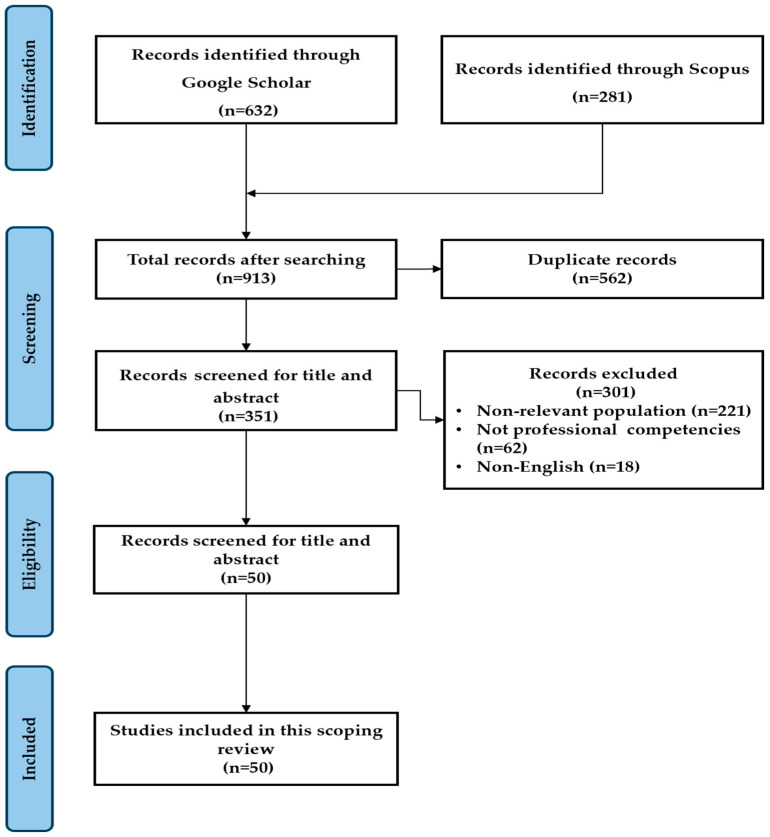
Flow chart of PRISMA process for a scoping review.

Following the title and abstract screening, the remaining 50 articles were subjected to full-text eligibility assessment. After applying the final inclusion and exclusion criteria, all 50 peer-reviewed articles were ultimately selected for this review (see the flow chart of the process in Figure 1).

### 3.2. Article Characteristics

The selected articles covered a wide range of geographical regions, with studies originating from America (*n* = 7), Europe (*n* = 9), and Asia (*n* = 13). Additionally, 21 studies did not specify a specific geographical location, reflecting a growing interest in the digital adaptation of older adults in different cultural contexts. Additionally, 10 articles were global systematic reviews or meta-analyses, which provided synthesized insights from multiple geographical regions.

Regarding methodological approaches, quantitative survey-based studies remained the most common (*n* = 23), followed by qualitative interview studies (*n* = 10) and mixed-method studies (*n* = 8). In addition, nine systematic reviews and meta-analyses were included due to their contribution to synthesizing empirical findings, identifying research gaps, and establishing theoretical frameworks relevant to technological proficiency and self-esteem. While most studies employed cross-sectional designs (*n* = 29) to assess technological proficiency and self-esteem at a single point in time, a subset of studies utilized longitudinal methods (*n* = 6) to track changes over time.

The articles were further classified based on their thematic focus. A total of 18 empirical studies specifically investigated the relationship between technological proficiency and self-esteem in older adults. Another 12 studies, including both empirical and review-based research, examined the barriers to digital engagement, highlighting factors such as age-related cognitive decline, lack of digital literacy programs, and economic disparities. Additionally, 11 systematic reviews and empirical studies focused on the psychological outcomes of technology use, addressing issues such as social isolation, autonomy, and mental well-being among older individuals (see the flow chart of the process in Table 1).

## 4. Discussion

### 4.1. Self-Esteem Trajectories

Self-esteem is regarded as a core concept within the field of psychology, representing a crucial part of psychological well-being. According to Rosenberg (1965), it is defined as a favorable or unfavorable attitude toward the self. Therefore, self-esteem embodies the appreciation of self-respect and self-worth in a person. It is regarded more as a global estimate than a domain-specific assessment of whether an individual is worthy or otherwise, as suggested by Jordan (2020). Though relatively stable across time, the dynamics of self-esteem are definitely expressed along the curve of life: the level is high in childhood, lowers during adolescence, rises in midlife, and lowers in the later stages of life, as indicated by Orth et al. (2018).

A developmental trajectory of self-esteem is one reflective of various age-specific challenges and accomplishments. In childhood, self-esteem is formed mainly through feedback from parents and peers (Minev et al., 2018). During adolescence, self-esteem often experiences ups and downs due to challenges posed by the formation of identity and social comparison (Krauss et al., 2020). Adulthood brings a reasonably high and stable degree of self-esteem, often augmented by the accomplishment of goals in life and a sense of personal accomplishment (Bleidorn et al., 2023). On the other hand, late adulthood brings decreased self-esteem, often occasioned by challenges related to retirement, health decline, and loss of social roles (Zhang et al., 2019). These forces may intensify feelings of obsolescence in a society that reveres both youth and technological skills (Šare et al., 2021).

In older adults, there are two interacting dimensions: self-worth and self-competence. According to Orth and Robins (2022), self-worth is the intrinsic value an individual considers themselves to possess regardless of extrinsic successes or others’ validation of the person. On the other hand, self-competence is defined as one’s belief in performing well in a particular task in order to reach one’s goals. According to Salice (2020), a sense of high self-competence often enhances self-worth in societies where personal productivity and achievement are highly valued. Major physical and social changes are experienced by older adults, thereby making these dimensions of self-esteem very prone to fluctuation.

Despite these, there are elements acting to temper this decline in self-esteem in older adults. In this regard, protective factors such as social support, financial security, and health all play their role in keeping self-esteem at higher levels for prolonged periods, according to von Soest et al. (2018). Technological proficiency has, thus, become an important new predictor that may enable older adults to respond more effectively to age-related threats to self-esteem. In this respect, promoting digital skills also increases the scope for social inclusion, autonomy, and competence, all of which are reinforcing factors for self-esteem, according to Wilson (2018).

The dynamic nature of self-esteem underlines the importance of age-specific factors in shaping psychological outcomes. Probably the most widely used measure in assessing global self-esteem is the Rosenberg Self-Esteem Scale, developed by Rosenberg (1965). Its ten items represent, in a condensed form, a balance between positively and negatively keyed items, thereby controlling for response biases such as acquiescence (Gnambs et al., 2018). This instrument shows very high reliability, with a Cronbach’s alpha ranging from 0.77 to 0.88, and good test/retest reliability (Monteiro et al., 2022). The simplicity and clarity of the RSES make this scale particularly suitable for older adults, especially for those who have some kind of cognitive or literacy problem (Mayordomo et al., 2020). However, though the RSES does cover global self-esteem well, it does not strongly represent such age-specific aspects as health, independence, or social role transitions, which might be most important among older adults (Ogihara & Kusumi, 2020). In fact, a lot of focused research is needed to understand self-esteem as a dynamic construct that unfolds across a life span.

### 4.2. Technological Proficiency: Influencing Factors

#### 4.2.1. Technological Proficiency and Aging

Technological proficiency, being a multidimensional construct, refers to the way one feels about and can use modern technologies. It has emerged as a key determinant of how well older adults adapt to modern life (Klimova & Poulova, 2018). Beyond its practical utility in daily living, technological proficiency profoundly impacts psychological well-being, including self-esteem. Casanova et al. (2021) undertook a systematic review of 11 studies on the usage of digital technology among older adults and how it contributes to well-being and perceptions of loneliness. The results reported a connection between the usage of digital technology and reduced well-being and loneliness among older adults. The understanding of the interconnection between technological proficiency and self-esteem among older adults is important because, as Y. Chen and Gao (2023) found, increased technological proficiency in the form of greater self-efficacy in the usage of digital tools had a notable influence on self-esteem by increasing information involvement and reducing loneliness in old age.

Some important implications of technological proficiency in an aging population include the frequency of technology use, proficiency level, and adaptability. These elements interact with personal and environmental factors as predictors of the potential for older adults to use digital tools and maintain psychological and social well-being. Low proficiency in these fields can increase feelings of isolation and exclusion among older adults because of a growing digital divide (Saubern et al., 2020).

Usage frequency, defined as how often an individual interacts with technology, can vary considerably based on factors such as physical constraints (e.g., reduced mobility or vision), access to devices, and the availability of social or familial support (Meng et al., 2022). Older adults usually face more barriers to frequent technology use, including physical challenges, decline in cognitive function, and lack of encouragement from their social environment. Skill, referring to the technical ability necessary to operate digital devices such as smartphones and computers, is important for accessing various essential services, including healthcare, financial management, and social networking sites. Lower skill levels, as found by Roque and Boot (2018), may prevent older adults from engaging in a digitized society and thus limit their independence and access to resources that can be useful.

Another critical factor that can be used to define technological proficiency is adaptability, which is the ability to be taught and incorporate a new technology into daily life. Indeed, most sudden changes in technology require adaptability, although this may be impaired in most older adults because of cognitive decline, unfamiliarity, and lack of training opportunities (Morgan et al., 2022). Adaptability in this age group holds a vital key to closing the digital gap because, through such ability, older adults can keep pace with technological changes and thereby have better control over some aspects of their lives.

The influence of technological proficiency goes beyond technical engagement and includes broader psychological and social outcomes. More competence is related to better psychological well-being because it decreases loneliness, depression, and anxiety (K. Kim et al., 2021). Digital communication tools, such as video calling platforms and social media, provide older adults with opportunities to maintain social connections and reduce isolation (Anderberg et al., 2019). Moreover, higher technological proficiency fosters self-efficacy and autonomy, which are important components of self-esteem in later life (Lozoya et al., 2022). However, as Y. Chen and Gao (2023) point out, the advantages of technological proficiency are not distributed equally, and factors such as gender, socioeconomic status, and educational background have a substantial impact on the accessibility and use of technology. Women, people from lower-income backgrounds, and less-educated individuals are among those most often hit hard by the digital divide, which worsens already-existing inequalities.

#### 4.2.2. Technology Acceptance Model

The technology acceptance model provides a theoretical framework for understanding how older adults use and feel about technology and factors that affect their proficiency. The TAM postulates that PU and PEOU are important variables behind attitudes toward technology and its adoption (Granić & Marangunić, 2019). However, for older adults, perceived ease of use may be negatively affected due to cognitive decline, loss of motor skills, or lack of experience with technology (Yap et al., 2022). With increases in their proficiency levels, the sense of self-efficacy among older adults rises, which promotes a sense of accomplishment and control that increases their self-esteem (Quialheiro et al., 2023).

**Figure 2:**
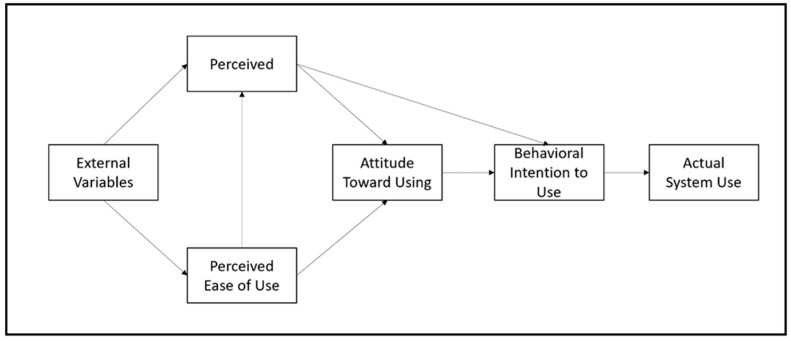
Technology acceptance model (Marangunić & Granić, 2015).

The additional social effect of technological engagement in terms of increased connectivity and lower isolation also reinforces the psychological benefits of technological proficiency. Technologies that alleviate social inclusion, for instance, video chatting and apps, can facilitate health monitoring and enable autonomy and independence, which are central to the psychological well-being of older adults (Cebreros-Valenzuela et al., 2020). Such tools provide functional advantages but also foster self-esteem by promoting a sense of personal control over daily tasks and health needs (see Figure 2).

### 4.3. Relationship Between Technological Proficiency and Mental Health

Technological proficiency shapes the mental health of older adults by influencing their ability to maintain social connections, autonomy, and psychological resilience in a digitized world. Greater ability in using modern technologies has been linked to lower feelings of loneliness, anxiety, and depression among older populations, since it helps them remain socially connected and access mental health resources and increases feelings of autonomy and competence (Shareef et al., 2021; Zhu et al., 2022).

Such digital support services help alleviate feelings of loneliness and enhance the levels of social connection among older adults, as observed by Naudé et al. (2022). In fact, studies performed by Bonsaksen et al. (2021) found that older adults who frequently use such digital aids have much higher levels of social integration and psychological well-being. Comparable results were described by Choi and Lee (2021): the use of ICT enhances psychological well-being, particularly when social support is strong or among the frail. Each of these findings suggests that the use of digital tools might serve to protect against unfavorable psychological outcomes associated with aging, especially feelings of loneliness and an overall sense of isolation.

Moreover, technological proficiency is closely associated with self-efficacy and autonomy, both of which are crucial elements of mental health and self-esteem in later life. The ability to use technologies, such as health-monitoring apps and wearable devices, empowers older adults to take care of their health independently, promoting a sense of control over their lives (Kulurkar et al., 2023). Increased autonomy and perceived competence stemming from technological engagement can also enhance psychological resilience, further promoting mental health (García et al., 2022).

However, technological proficiency does not equally benefit all older adults. The digital divide exacerbates existing inequalities, created by disparities in socioeconomic status, educational background, and gender, and can be detrimental to the mental health of less-skilled individuals (Mubarak & Suomi, 2022). Older adults who are not good at using technologies often report frustration, anxiety, and inadequacy, leading to social withdrawal and decreased mental health (Holgersson & Söderström, 2019). For instance, people with limited exposure to or training in technology may feel themselves excluded from a rapidly digitizing society, which further exacerbates feelings of loneliness (Tsertsidis et al., 2019).

The technology acceptance model, mentioned above, and the unified theory of acceptance and use of technology provide valuable insights into the psychological mechanisms explaining the role of technological proficiency in mental health. The TAM stipulates that perceived ease of use and usefulness will contribute to older adults’ engagement with technology, hence impacting their mental health (J. Chen et al., 2023; Zin et al., 2023). The UTAUT takes this a step further by highlighting social influence and facilitating conditions as key determinants of forming technology adoption behavior. In fact, the theoretical framework is based on the notion that the better the supportive environments and user-friendliness of the interfaces, the better the outcomes of technological engagement in terms of mental health (Palas et al., 2022).

Interventions aimed at overcoming this problem of poor technological savvy among older adults include community-based technology training programs, intergenerational learning models, and the development of simplified user interfaces. These interventions have been advocated for in the literature to date. Indeed, Awan et al. (2021) and Jokisch et al. (2020) suggest that these kinds of initiatives may provide a way to decrease the digital divide while promoting digital inclusion, which has the consequent benefit of improving mental health. Program participants are thus better prepared, and their skills and confidence in using digital technologies have been found to alleviate possible feelings of frustration and exclusion, further benefiting overall psychological health.

### 4.4. Relationship Between Technological Proficiency and Self-Esteem

Various perspectives on the psychological and social meaning of the relationship between technological proficiency and self-esteem have been elicited from older adults in research targeting this age group. Lee et al. (2022) conducted research on how digital technology enhances the social connectedness of older individuals; the findings indicate that habitual use increases feelings of connectedness and therefore indirectly encourages self-esteem. Boлoшинa et al. (2022) also showed that the use of information and communication technologies contributes to positive psychological welfare, implying that increased social interaction from the use of technology boosts self-esteem.

Pan argued that technological proficiency is very significant in enhancing self-efficacy, which is a central part of self-esteem. The author of this study concluded that the more familiar an older person becomes with modern technologies, the higher his/her self-esteem will be because of enhanced feelings of competence and mastery. Zander et al. (2020) explained that technology practice enhances independence, whereby older adults become able to take care of their health and activities of daily living by themselves; as such, their self-esteem is enhanced.

By contrast, Özsungur (2022) discussed the challenges of low technological skills in older persons; for them, every technological problem normally creates frustration and leads to a decrease in self-esteem. The findings of the study are in line with a study by Parsakia (2023), who found that poor adoption and use of technology may become a barrier to social participation and autonomy, causing further reduction in self-esteem. Czaja et al. (2018) pointed out that digital engagement may also enhance cognitive functioning and social interaction, hence indirectly providing support for psychological resilience and self-esteem.

Regarding psychological benefits, Poscia et al. (2018) indicated an increase in self-efficacy and autonomy, two relevant aspects of self-esteem. Most of these works, such as the studies by Llorente-Barroso et al. (2021) and Shareef et al. (2021), did not deal directly with the relationship between technological skills and self-esteem, but rather with general well-being. However, assuming good results obtained for social inclusion and cognitive stimulation derived from technology, one could predict a positive relationship with self-esteem.

Wang et al. (2023) carried out a quasi-experimental study on how a technology-based cognitive stimulation program enhanced self-esteem, self-efficacy, and autonomy in older adults. This provides some evidence for the notion that technological engagement provides positive psychological outcomes of self-esteem, as improvement in cognitive and social abilities bolsters such self-concepts.

## 5. Conclusions

The reviewed literature describes a complex interrelationship between technological proficiency and self-esteem in older age, with both positive and negative implications. Over the years, research has increasingly emphasized the role of technological proficiency in psychological functioning, particularly in how it is associated with a person’s level of self-efficacy, connectedness with others, and autonomy. Empirical research suggests that a greater level of competency in the usage of digital technology is associated with increased self-esteem because technological proficiency creates a perception of competency and a state of having control over one’s life. Iancu and Iancu (2020), for instance, reported that older adults with high technological proficiency reported high levels of societal participation and self-efficacy, key elements in psychological resilience. Likewise, research conducted by Nguyen and Le (2021) and Rocha et al. (2021) has proven how technology such as video calling and social media helps older adults maintain and expand their connections with other people and mitigate the perception of disconnection and a state of not belonging. The ability to master technology helps in not only staying connected but also psychological functioning because technological proficiency produces a perception of accomplishment and a state of having mastered life.

In addition to social interaction, the use of digital services, such as telehealth and online banking, boosts self-esteem because these services enable older adults to independently manage their health and financial situations. Mubarak and Suomi (2022) posit that this ability boosts self-efficacy, which in turn results in a boost in self-esteem. Mastery and perceived control are of particular importance in this context, as they instill confidence in older adults as they navigate an increasingly digital world.

Nonetheless, a shortage in technological proficiency is a negative influencing factor on self-esteem. A significant percentage of older adults suffer frustration and lower self-esteem while trying to grasp and handle digital technologies. In accordance with the hypothesis provided in Asad et al. (2023), technology-related problems are a key source of exclusion, learned helplessness, and solitude. Likewise, technology anxiety is a major source emphasized in H. N. Kim et al. (2023), increasing psychological vulnerability and lowering self-esteem. In addition, these problems are inextricably tied with socioeconomic disadvantages, level of education, and access barriers. Zhu et al. (2022) noted that low-resource older adults are disproportionately burdened with increased social and psychological disadvantages.

From a social perspective, these findings emphasize the need to address the digital divide in order to promote greater inclusion and psychological health among older people. Gerontologists, psychologists, and technological innovators need to work together to design interventions aimed at enhancing technological skills and reducing digital anxiety. It is also critical that government agencies and policymakers prioritize digital literacy programs targeting older people to provide equal access to technology and counteract the negative consequences related to digital exclusion.

Notwithstanding these results, the literature presents a number of significant limitations. Firstly, a significant number of research studies on social inclusion and happiness were conducted in a heterogenous and imprecise way, without focusing on self-esteem as a key outcome variable. Secondly, the overreliance on cross-sectional approaches limits the ability to identify whether technological ability operates as a causal variable or only a correlational dimension of self-esteem. In addition, the reliance on self-reported tools risks inducing potential biases, such as desirability and memory biases, and undermines the validity of the results. Lastly, there is a shortage of research on how demographic variables, such as ethnicity, income level, and geographic area, relate to technology usage and psychological results.

To examine the gaps identified in this review, future research should engage in longitudinal approaches to track evolving dynamics between technological proficiency and self-esteem over a longer timescale, particularly among aging groups facing challenges with rapid technological advancement. Widening sample groups to represent a diverse population will maximize generalizability and reveal situational variable effects on these dynamics. By overcoming these limitations, future research will gain important insights into the psychological impact of technological proficiency among aging groups, which can facilitate the development and implementation of targeted interventions aimed at helping older adults in a rapidly digitizing world.

## Figures and Tables

**Table 1:** Characteristics of included articles.

Author(s) and Years	Country	Study Design	Sample	Instrument(s)
Awan et al. (2021)	Pakistan	Review study	Older adults using smartphones	Systematic review and AHP analysis
Barbosa Neves et al. (2019)	Multiple (international)	Quantitative study	12 older adults in residential care	Semi-structured interviews, psychometric scales, field observations, and usability tests
Bonsaksen et al. (2021)	Norway, UK, USA, and Australia	Quantitative study	836 older adults (60–69 years and 70+ years)	Online survey and multiple regression analysis
Casanova et al. (2021)	Multiple (international)	Review study	Older adults using ICT and SNS	Systematic review, and thematic and content analyses
Cebreros-Valenzuela et al. (2020)	Mexico	Quantitative study	63 older adults in three senior day-care centers	Cognitive stimulation program (PESCO MX) and comparison with non-technological recreational activities
J. Chen et al. (2023)	China	Quantitative study	Older adult users	Partial least square regression and structural equation modeling
Y. Chen and Gao (2023)	China	Quantitative study	276 older Chinese adults (aged 60–90)	Social media self-efficacy scale and informational use analysis
Choi and Lee (2021)	South Korea	Review study	Older adults	Literature review and data extraction from 3 databases (Ovid-Medline, Ovid-EMBASE, and Cochrane library)
Czaja et al. (2018)	USA	Quantitative study	300 older adults (M_age_ = 76.15 years) at risk of social isolation	PRISM system vs. control group (binder condition)
García et al. (2022)	Spain	Qualitative study	Older adults (65+ years) in Spain	Semi-structured interviews and phenomenological perspective
Gnambs et al. (2018)	Not specified	Quantitative study	113 independent samples (N = 140,671)	Rosenberg Self-Esteem Scale (RSES)
Holgersson and Söderström (2019)	Sweden	Quantitative study	Older adult citizens	Survey and qualitative categorization
Iancu and Iancu (2020)	Not specified	Qualitative study	Older adult individuals	Review of design principles for older adult-friendly technology
Jokisch et al. (2020)	Germany	Quantitative study	131 older technology experts and 239 non-experts	General Internet Self-Efficacy (GISE), Communication Internet Self-Efficacy (CISE), and structural equation modeling
H. N. Kim et al. (2023)	Not specified	Review study	Older adults	Systematic literature review using PRISMA standards
K. Kim et al. (2021)	South Korea	Quantitative study	264 older adult smartphone users	Survey on smartphone proficiency, use, loneliness, and ego integrity
Klimova and Poulova (2018)	USA	Review study	Older adults	Review of technology acceptance models
Krauss et al. (2020)	USA (Mexican-origin families)	Quantitative study	674 families (children aged 10–16)	Multi-informant reports (parents and children) and cross-lagged panel models (CLPM and RI-CLPM)
Kulurkar et al. (2023)	Not specified	Quantitative study	Older adult individuals	Wearable sensors, AI-based IoT system
Lee et al. (2022)	South Korea	Quantitative study	50 community-dwelling older adults	Intergenerational Forum (IF) program and multiple outcome measures
Llorente-Barroso et al. (2021)	Not specified	Qualitative study	Older adults (60+ years) and experts in older adult education	Focus groups and in-depth interviews
Lozoya et al. (2022)	Not specified	Quantitative study	380 retired older adults	ICT usage scale and self-efficacy scale
Meng et al. (2022)	Not specified	Quantitative study	232 older adult users	Technology anxiety, health anxiety, and affective and cognitive trust measures
Minev et al. (2018)	Bulgaria	Quantitative study	40 adolescents (20 boys and 20 girls, aged 14)	Self-Esteem Scale (RSE)
Morgan et al. (2022)	Not specified	Quantitative study	324 higher education students	Online survey and digital literacy assessment
Mubarak and Suomi (2022)	Not specified	Review study	Older adults population	Review of digital exclusion literature
Naudé et al. (2022)	Not specified	Review study	Older adults in older adult care institutions	Video call implementation studies
Ogihara and Kusumi (2020)	Japan	Quantitative study	6113 participants (aged 16–88)	Rosenberg Self-Esteem Scale
Orth and Robins (2022)	Not specified	Review study	Multiple samples	Review of self-esteem studies
Özsungur (2022)	Turkey	Quantitative study	687 older adults individuals	Successful aging scale and technology acceptance model (UTAUT2)
Palas et al. (2022)	Bangladesh	Qualitative study	493 older adults individuals (60+ years)	Extended UTAUT2 model (Service Quality and Quality of Life)
Pan (2020)	China	Quantitative study	332 undergraduate students	Technology acceptance, self-efficacy, and learning motivation questionnaires
Parsakia (2023)	Not specified	Review study	Students in educational settings	Chatbot and AI impact studies
Poscia et al. (2018)	Not specified	Review study	Older individuals	Review of loneliness intervention studies
Quialheiro et al. (2023)	Portugal	Quantitative study	120 middle-aged and older adults (55+ years)	Workshops for Online Technological Inclusion (OITO) project and Mobile Device Proficiency Questionnaire
Rocha et al. (2021)	Not specified	Review study	General population	Review of social media misinformation studies
Roque and Boot (2018)	Not specified	Quantitative study	Older adults	Mobile Device Proficiency Questionnaire (MDPQ and MDPQ-16)
Salice (2020)	Not specified	Qualitative study	General population	Conceptual analysis of self-esteem and social esteem
Šare et al. (2021)	Croatia	Quantitative study	Older adults in nursing homes	Rosenberg Self-Esteem Scale, Patient Health Questionnaire, and Generalized Anxiety Disorder Scale
Saubern et al. (2020)	Not specified	Qualitative study	Teachers	TPACK Confidence and TPACK Usefulness assessment
Shareef et al. (2021)	Canada	Quantitative study	179 older adults and disabled individuals in retirement homes and rehabilitation centers	Trust disposition model, structured experiments, and perception survey
Tsertsidis et al. (2019)	Multiple (Western Europe, USA, Australia, Taiwan)	Review study	Seniors aged 60+ in home environments	Analysis of post-implementation technology acceptance studies
von Soest et al. (2018)	Norway	Quantitative study	5555 adults (M_age_ = 58 years)	Self-esteem, personality traits, and socioeconomic and health data
Wang et al. (2023)	China	Quantitative study	394 rural older adult individuals	Psychological Digital Divide (PDD) model and Relative Deprivation Theory (RDT)
Yap et al. (2022)	Not specified	Quantitative study	Older adults	Capability Approach and Technology Acceptance Model
Zander et al. (2020)	Not specified	Review study	Older individuals using welfare technology	Review of evaluation methods for welfare technology
Zhang et al. (2019)	China	Quantitative study	283 older adults	Meaning in Life Questionnaire, Rosenberg Self-Esteem Scale, and Death Anxiety Scale
Zhu et al. (2022)	Not specified	Review study	Studies on smart home older adult care ethics	Review of ethical concerns in AI-enabled older adult care
Zin et al. (2023)	South Korea	Quantitative study	170 Korean older adults	Extended Technology Acceptance Model (TAM)
Boлoшинa et al. (2022)	Not specified	Quantitative study	78 older adults individuals (65–72 years)	Psychodiagnostic techniques and SPSS 21.0 analysis

## Data Availability

No new data were created or analyzed in this study. Data sharing is not applicable to this article.
